# Metabolomics of neurological disorders in India

**DOI:** 10.1002/ansa.202000169

**Published:** 2021-11-28

**Authors:** Sangeetha Gupta, Uma Sharma

**Affiliations:** ^1^ Amity Institute of Pharmacy Amity University Noida Uttar Pradesh India; ^2^ Department of NMR & MRI Facility All India Institute of Medical Sciences New Delhi India

**Keywords:** GC‐ MS, Indian perspective, LC‐MS, metabolites, metabolomics, neurological disorders, nuclear magnetic resonance (NMR)

## Abstract

Metabolomics is the comprehensive study of the metabolome and its alterations within biological fluids and tissues. Over the years, applications of metabolomics have been explored in several areas, including personalised medicine in diseases, metabolome‐wide association studies (MWAS), pharmacometabolomics and in combination with other branches of omics such as proteomics, transcriptomics and genomics. Mass spectrometry (MS) and nuclear magnetic resonance (NMR) spectroscopy are the major analytical techniques widely employed in metabolomics. In addition, MS is coupled with chromatography techniques like gas chromatography (GC) and liquid chromatography (LC) to separate metabolites before analysis. These analytical techniques have made possible identification and quantification of large numbers of metabolites, encompassing characterization of diseases and facilitating a systematic and rational therapeutic strategy based on metabolic patterns. In recent years, the metabolomics approach has been used to obtain a deeper insight into the underlying biochemistry of neurodegenerative disorders and the discovery of biomarkers of clinical implications. The current review mainly focuses on an Indian perspective of metabolomics for the identification of metabolites and metabolic alterations serving as potential diagnostic biomarkers for neurological diseases including acute spinal cord injury, amyotrophic lateral sclerosis, tethered cord syndrome, spina bifida, stroke, Parkinson's disease, glioblastoma and neurological disorders with inborn errors of metabolism.

**Abbreviations**
ALS: Amyotrophic lateral sclerosis
ASCI: Acute spinal cord injury
CPMG: Carr–Purcell Meiboom–Gill
CSF: cerebrospinal fluid
DOPAC: 3,4‐Dihydroxyphenylacetic acid
GABA: γ‐aminobutyric acid
GBM: Glioblastoma multiforme
GC‐MS: gas chromatography‐mass spectrometry
HG: high‐grade
HC: healthy controls
IEM: Inborn errors of metabolism
IPD: idiopathic Parkinson's disease
LC‐MS: liquid chromatography‐mass spectrometry
LC‐MS/MS: liquid chromatography‐tandem mass spectrometry
LG: low‐grade
LMMC: lipomeningomyelocele
MCAo: middle cerebral artery occlusion
MMC: meningomyelocele
MoM: multiples of median
MS: mass spectrometry
MSA: multiple system atrophy
MSUD: Maple syrup urine disorder
NMR: nuclear magnetic resonance
OPLS‐DA: Orthogonal Partial Least Square‐Discriminant Analysis
PCA: Principal component analysis
PD: Parkinson's disease
PLS‐DA: partial least squares discriminant analysis
PQ: Paraquat
PSP: progressive supranuclear palsy
PTUPB: (4‐(5‐phenyl‐3‐3‐3‐(4‐ trifluoromethyl‐phenyl)‐ureido‐propyl‐pyrazole‐1‐yl)‐benzenesulfonamide)
ROS: Reactive oxygen species
ROT: rotenone
SCI: spinal cord injury
VIP: variable importance in projection

## 1 INTRODUCTION

Neurological disorders comprise abnormal conditions related to the central nervous system and peripheral nervous system predominantly including dementias, migraine, epilepsy, Alzheimer's disease, stroke, neuroinfectious conditions (encephalitis, tetanus, meningitis), multiple sclerosis, Parkinson's disease, brain tumours, traumatic brain injury, motor neuron diseases and neurological deficits as a result of malnutrition and exposure to neurotoxic substances.^1^ Globally, neurological disorders stand as the second leading cause of death after cardiac diseases, contributing to approximately 9 million deaths, almost 16.5% of total mortalities.^2^ Neurological disorders are also one of the leading causes of disability, affecting 276 million people globally, contributing to 11.6% of disability‐adjusted life years.^3^ Worldwide, more than 6 million people die because of stroke each year; more than 50 million people have epilepsy, 47.5 million people have dementia, 7.7 million new cases annually reported for Alzheimer's disease and more than 10% is the prevalence of migraine.^4^

In India, the prevalence of neurological disorders ranges from 967 to 4070 with a mean of 2394 persons per 1,00,000 population. It contributes to approximately 20 to 30 million people suffering solely from various forms of neurological disorders.^5^ The prevalence of neurological disorders in India is higher in rural areas as compared to urban areas.^6^ The prevalence rate of stroke in tribal or rural areas is 109 per 1,00,000 populations, while in the urban areas is 52 to 472 per 1,00,000 populations. Similarly, the prevalence rate of epilepsy in rural areas is 2.5 per 1000 populations, while in urban areas is 2.5 to 11.9 per 1000 populations.^6^, ^7^ According to the 2001 census in India, all age groups are vulnerable and affected by one or other neurological disorders. However, the peak was observed explicitly in people above 60 years of age. Thus, as 77 million people were above 60 years of age in 2001, the numbers were projected to increase to 177 million by 2025, contributing significantly to age‐associated disorders, particularly for Parkinson's disease, dementia and cerebrovascular disorders like stroke.^7^, ^8^ In India, there has been a 44% increase in mental, neurological and substance use disorders from 1990 to 2013. It has been speculated that if the same trend continues, it may increase further to 23% by 2025, which would be a dreadful situation for neurologists.^9^

The treatment of neurodegenerative diseases is challenging as establishing a quantitative and objective diagnostic criterion is difficult. Metabolomics is a well‐studied branch of “omics” which encompasses the identification, characterization and analysis of endogenous metabolites in biofluids/tissues of biological systems.^10^ Metabolomics is an integral approach of systems biology, providing information on various biochemical pathways mediated through small molecules known as metabolites. Metabolomics acts as a connecting link between genotypic and phenotypic profiles for complex diseases such as neurological disorders.^11^ This approach generates “Big Data,” which requires extensive data processing such as algorithmic peak detection, peak alignment and annotation of putative compounds along with computationally intensive analyses for the classification of groups.^12^

Metabolome identification and interpretation involve a series of different analytical complementary methods.^13^ Two principal analytical techniques commonly used in metabolomics are mass spectrometry (MS) coupled to chromatographic techniques and nuclear magnetic resonance (NMR) spectroscopy.^14^ The MS‐coupled chromatographic techniques widely implicated for the analysis of biological samples are: gas chromatography‐mass spectrometry (GC‐MS), liquid chromatography‐mass spectrometry (LC‐MS), gas chromatography‐tandem mass spectrometry (GC‐MS/MS), liquid chromatography‐tandem mass spectrometry (LC‐MS/MS), ultra‐performance liquid chromatography‐mass spectrometry and capillary electrophoresis‐mass spectrometry.^15^ The MS provides advantages of assessment of both volatile and non‐volatile compounds. The GC‐MS technique efficiently allows the separation, identification and quantification of semi‐volatile and volatile compounds. At the same time, LC‐MS is particularly suitable for analysing thermally unstable and non‐volatile compounds in biological fluids.^16^ However, the limitation of LC‐MS is the no availability of standard spectral libraries, such as NIST and Wiley, compared to the GC‐MS.^17^

NMR spectroscopy is a highly reproducible, high‐throughput and non‐invasive tool for structural determination and quantitative analyses of metabolites in vitro and in vivo.^18^, ^19^ Proton nuclear magnetic resonance spectroscopy (^1^H NMR) has been widely used for metabolomics due to its robust nature, natural abundance and minimal sample preparation.^20^, ^21^, ^22^, ^23^, ^24^ The major disadvantage of NMR spectroscopy is its lower sensitivity compared to MS‐based techniques, with its detection limits ranging between micromolar to sub‐micromolar levels. However, recent developments in cryoprobes, nanoprobes and high magnetic field spectrometers have increased the sensitivity and resolution of NMR, increasing the number of detectable metabolites.^25^, ^26^, ^27^, ^28^ Despite these developments, the MS‐based metabolomics applications outnumber the NMR‐based techniques as the number of detectable metabolites through MS ranges from 300 to 1000 plus, which is significantly higher than NMR spectroscopy. In addition, coupling chromatography techniques with MS allows the detection of secondary metabolites even at lower concentrations. Furthermore, MS‐based analysis requires a small sample volume (as low as 10 µL) compared to NMR spectroscopy. The cheaper instrument cost is another advantage of MS‐based analysis over the NMR‐based metabolomics approach.^15^, ^16^, ^17^, ^18^, ^19^, ^20^, ^21^, ^22^, ^23^, ^24^, ^25^, ^26^, ^27^, ^28^

Metabolomic strategies cover two approaches “untargeted discovery global” and “targeted validation tandem.”^14^, ^29^ Untargeted discovery metabolomics is based on a hypothesis‐generating approach. In contrast, targeted metabolomics includes hypothesis testing for validation of untargeted analysis. Thus, in the untargeted discovery of metabolomics information on metabolome, identification of pattern and “metabolic fingerprinting” are performed to classify phenotypes and their associated interaction pathways.^30^ On the other hand, in targeted metabolomics approaches, also known as “biased or directed metabolomics” or “metabolic profiling,” a quantitative analysis of known or targeted metabolites is performed to explore perturbations in metabolic pathways and classification of phenotypes.^31^ Thus, the metabolomics approach has a broad scope in establishing diagnostic markers of various neurodegenerative diseases and, thus, has the potential for a more personalised therapeutic approach. Over the years, metabolomics has evolved as a dynamic research technique uncovering pathogenic mechanistic pathways through the identification of novel biomarkers for neurological disorders.^32^

In this review, the studies reported from India using the metabolomics approach using NMR spectroscopy and MS analysis, whichever has been used in neurological diseases are described in detail. These studies include both clinical and animal studies exploring metabolic alterations in various neurological disorders. Search engines used were PubMed and Google Scholar databases. The search strategy included the following keywords: (Neurological disorders AND India AND Metabolomics), (Acute spinal cord injury/Amyotrophic lateral sclerosis/Tethered cord syndrome/Spina bifida/Stroke/Parkinson's disease/Glioblastoma/ Neurological disorders with inborn errors of metabolism AND India AND Metabolomics). The inclusion criteria for studies were: Research articles either on clinical or animal models of neurological disorders evaluated in India. The exclusion criteria were: neurological disorders evaluated in India apart from the metabolomics approach, neurological disorders evaluated apart from India using the metabolomics approach and review articles.

## 2 METABOLOMICS OF NEUROLOGICAL DISORDERS IN INDIA

**FIGURE 1:**
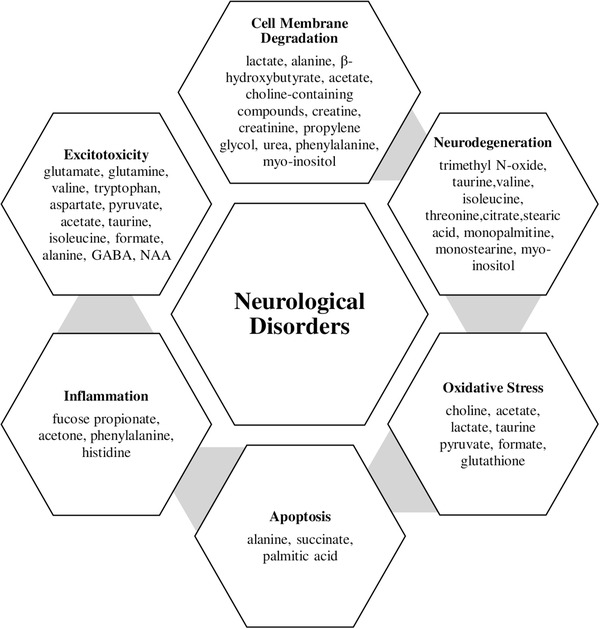
Flow chart representing the role of neurometabolites in the pathophysiology of various neurological disorders. GABA: γ‐aminobutyric acid; NAA: *N*‐aspartyl aspartate

The studies related to metabolomics of neurological disorders in India, including acute spinal cord injury (SCI), amyotrophic lateral sclerosis, tethered cord syndrome, spina bifida, stroke, Parkinson's disease, glioblastoma and neurological disorders with inborn metabolic abnormalities, have been elaborated as follows. Each condition is explained with its pathophysiology and prevalence, followed by metabolomics studies performed in India with experimental details. The pathophysiological mechanisms and role of neurometabolites in neurological disorders have been summarised in Figure 1 and Table 1.

**TABLE 1:** Summary of the metabolomic studies of neurological disorders in India

Neurological Disorders	References	Biological sample/sample size	Type of study/geographical location	Metabolic alterations Increased Decreased		Conclusions
Acute spinal cord injury	[^39^, ^40^]	Serum analysed by NMR in 30 subjects Urine analysed by NMR in 140 subjects	Prospective case control pilot study/Lucknow, India Prospective case control study/Lucknow, India	Succinate, acetate, acetone, isoleucine, lactate, alanine, β‐hydroxybutyrate, creatine, creatinine, creatine phosphate, glucose, phenylalanine, propylene glycol, urea, 3‐ methylhistidine, glutamine	Valine, glycine	Activation of calcium‐dependent ionic channels; metabolic inefficiency; ischemic hypoperfusion; hypermetabolic Krebs cycle; altered glycolysis, amino acid metabolism, urea cycle and ATP depletion
Amyotrophic lateral sclerosis	[^46^]	Serum analysed by NMR in 40 subjects	Case control study/Lucknow, India	Glutamate, β‐hydroxybutyrate, acetate, acetone, formate	Glutamine, histidine	Increased oxidative stress, excitotoxicity, ATP depletion, metabolic acidosis, enhanced ketone bodies levels, motor abnormalities
Tethered cord syndrome	[^62^]	Cerebral spinal fluid analysed by NMR in 16 subjects	Case control study/New Delhi, India	Lactate, choline, glycerophosphorylcholine, acetate, alanine	−	Increased oxidative stress, mitochondrial dysfunction, anaerobic respiration
Spina bifida	[^74^]	Cerebral spinal fluid analysed by NMR in 16 subjects	Case control study/New Delhi, India	Lactate, choline, glycerophosphorylcholine, acetate, alanine	−	Neuronal dysfunction, anaerobic respiration, hypoxia, oxidative stress
Stroke	[^87^, ^88^, ^89^]	Rat brain cortex analysed by NMR in 21, 17 and 39 male Wistar rats, respectively	Experimental (middle cerebral artery occlusion model of transient focal cerebral ischemia)/New Delhi, India	Tyrosine, alanine, valine, acetate, lactate	Adenosine‐diphosphate, taurine, myoinositol, NAA, glutamate, glutamine, γ‐amino butyric acid, myoinositol, purine and pyrimidine metabolites, aspartate, choline, creatine/phosphocreatine, glycerophosphorylcholine	Oxidative stress, neuroinflammation, excitotoxicity, mitochondrial dysfunction, altered amino acid metabolism, nucleotide metabolism, cell death
Parkinson's disease	[^92^, ^93^, ^94^, ^95^, ^96^]	Saliva, urine and serum samples analysed by NMR in 113, 150 and 52 subjects, respectively. Brain of *Drosophila melanogaster* analysed by GC‐MS and LC‐MS/MS method	Case control study/New Delhi, India^81^, ^82^ Retrospective case control study/Lucknow, India⁸^3^ Experimental study/Lucknow and Tamil Nadu, India, respectively	Trimethylamine *N*‐oxide, GABA, *N*‐acetyl glutamate, acetoin, glutamine, lactate, phenylalanine, tyrosine, histidine, glycine, fucose, propionate, acetoacetate, taurine, valine, isoleucine, alanine, acetate, ornithine, formate −	− Dopamine	Altered neurotransmitters, gut microflora, energy metabolism, neuronal excitability, oxidative stress, dopaminergic neurodegeneration
Glioblastoma	[^99^]	Glial tumour samples of 13 patients and 3 control patients analysed by NMR	Case control study/Chennai, Tamil Nadu, India	Taurine, lactate, glutamine, glutamate, glycerol phosphocholine, valine, isoleucine, choline, tyrosine, leucine	Creatine, GABA, phenylalanine, l‐myoinositol, glutathione	Pattern of metabolic alterations was found be consistent with specific grade of glioblastoma
Inborn errors of metabolism	[^107^, ^109^, ^112^]	Dried blood spot samples by tandem mass spectrometry and urine by GC‐MS in a 17‐day old new born Dried blood spot samples by tandem mass spectrometry 4‐day‐old new born Dried blood spot: glutaryl carnitine (C5DC) and C5DC /C16 ratio by tandem mass spectrometry Urine: 3‐hydroxylglutaric acid and glutaconic acid by GC–MS/MS	Case report/Kelambakkkam. Tamil Nadu, India Case report/Durgapur, West Bengal, India Retrospective study/samples collected from all over the country	Blood: leucine/isoleucine/hydroxyproline, valine Urinary excretion: isovaleric acid, allo‐isoleucine‐2, isoleucine‐2, leucine‐2, valine‐2 Citrulline, glycine Blood: glutaryl carnitine (C5DC) and C5DC/C16 ratio Urinary excretion: 3‐hydroxylglutaric acid, glutaconic acid	− Arginine	Altered levels of neurotransmitters if untreated may lead to the development of severe encephalopathy in maple syrup urine disorder Citrullinemia, glycinaemia with hyper‐ammonaemia along with developed generalised tonic‐clonic seizures were observed Tandem mass spectrometry was found to be more than 93.33% sensitive and more than 99.42% specific for analysis metabolite levels in Glutaric acidemia type I (GA1) disorder

### 2.1 Acute SCI

The spinal cord comprises nerves that create communication links between the brain and other body parts. The bruise or tear in the spinal cord leads to SCI. The disease may cause death in adults as well as in children. Mainly, it is of two types: primary SCI and secondary SCI.^33^ Primary injury comprises a blemish of the spinal cord; generally, it happens with failure of biochemical integrity and enhancement of the compression force to the spinal cord, resulting in neuronal degeneration, rupturing of blood vessels and cell membrane impairment. Whereas the physiological cascade of primary injury triggers the injury to the secondary phase.^34^ Acute SCI (ASCI) is a traumatic incidence comprising both primary phases followed by a secondary phase leading to the initiation of a series of pathophysiological events.^35^ Within a few minutes, the microvasculature ruptures from primary injury, leading to oedema and haemorrhage, thereby impairing the blood perfusion to the traumatised spinal cord. This further causes vasospasm and thrombosis, intensifying the ischemic injury. The intensified ischemia is interlinked with the glutamate release mediated ionic dysregulation through Na^+^ and Ca^2+^ influx and ultimately excitotoxicity.^36^, ^37^ The prevalence of SCI in India is 0.15 million, with an average annual incidence of 15,000 patients per year (Rehabilitation Council of India).^38^

Singh et al. explored the importance of metabolomic biomarkers in the diagnosis of ASCI by analysing the serum samples at baseline and after 6 months follow‐up by NMR spectroscopy. In this prospective case‐control pilot study, 30 subjects were included and were divided into three groups: Group 1 was comprised of ASCI subjects which were intervened by a conventional method as posterior instrumentation with titanium pedicle screw and rod system; Group 2 was comprised of ASCI subjects intervened by posterior instrumentation along with autologous bone marrow‐derived mononuclear cells rich in stem cells and Group 0 was comprised of a control group without pathologies. The one‐dimensional ^1^H NMR spectra at 800 MHz were acquired using Carr–Purcell Meiboom–Gill (CPMG) pulse with water pre‐saturation at 300 K. The experimental parameters used were: spectral width = 12,820.5 Hz, relaxation delay = 5.0 s and data points = 64 K. The spectra were calibrated through standard metabolite NMR spectra accessible in biological magnetic resonance bank (www.bmrb.wisc.edu) via NMR suite 8.1 (Chenomx) software and human metabolome database (www.hmdb.ca). Both univariate and multivariate analysis approaches were used for the analysis. Orthogonal Partial Least Square‐Discriminant Analysis (OPLS‐DA) and orthogonal signal correction‐principal component analysis were used to differentiate groups and identify significant metabolites. Out of 280 metabolites identified, 15 (acetone, acetate, alanine, glutamine, formate, glucose, isoleucine, threonine, glycine, histidine, lactate, succinate, phenylalanine, valine and tyrosine) were well resolved and were quantified. The results revealed that out of 15 metabolites, seven metabolites (glycine, acetone, isoleucine, succinate, lactate, acetate and valine) were found to be significantly altered. Further, out of these seven metabolites, five metabolites (glycine, acetone, isoleucine, succinate and lactate) were found to be significantly elevated (*p* < 0.05) from baseline to 6 months in comparison to Groups 0 versus 1, 0 versus 2 and 1 versus 2. The authors explained the alterations in these five metabolites among the three groups, which are discussed as follows.

In Group 0, no change in the levels of the above metabolites was observed after 6 months compared to baseline. The baseline levels of metabolites in Groups 1 and 2 as compared to Group 0 indicated elevated levels due to injury. In the present study, after 6 months, glycine levels were significantly elevated (*p* < 0.03) in Group 1 compared to Group 0. While the levels of glycine were found to significantly decline (*p* < 0.022) after 6 months in Group 2 compared to Group 0. Glycine plays a major role in the maintenance of muscle tone, its release in nerves is regulated by calcium‐dependent channels, while its reuptake by Na^+^/Cl^−^ dependent glycine transporters. During normal conditions, the glycine levels were low, while in ASCI, destruction of channels and transporters causes a significant elevation in its levels.

In Group 2, the acetone levels were significantly elevated (*p* < 0.02) after 6 months of treatment with stem cells compared to Group 0, indicating the improvement after intervention. In contrast, the acetone levels were significantly reduced (*p* < 0.04) after 6 months in Group 1 compared to Group 2. It may be attributed to how ketone bodies, such as acetone, serve as an alternative energy source during SCI. Thus, during ASCI, the acetone levels were lower as being consumed to provide energy for survival of injured parts depicting its neuroprotective activity. On the other hand, with improvement in ASCI condition, the acetone levels were elevated.

Isoleucine levels at baseline were elevated in Group 1 (2.62 ± 1.17 mg/dL) and Group 2 (8.20 ± 11.96 mg/dL) as compared to Group 0 (1.77 ± 0.46 mg/dL), indicating that isoleucine plays an important role in ASCI. After 6 months, isoleucine levels were significantly increased in Group 2 (3.74 ± 1.56 mg/dL) compared to Group 0 (1.98 ± 0.40 mg/dL) and Group 1 (2.09 ± 0.98 mg/dL) (*p* < 0.006), indicating that stem cell intervention ameliorated the condition by maintaining the isoleucine level. Isoleucine is a branched‐chain amino acid required to produce the neurotransmitters glutamate and γ‐aminobutyric acid (GABA). During SCI, increased demand for de novo glutamate synthesis might have led to increased isoleucine utilization. Changes in the isoleucine levels are positively correlated with recovery.

After 6 months of treatment with stem cells, the succinate levels were significantly elevated (*p* < 0.006) in Group 2 compared to Group 0, indicating stem cell intervention has improved the ASCI condition. Succinate has been shown to play a pivotal role in gene expression, and changes in its levels contributed to cell death and injury. During ASCI, there is an excessive generation of enzymes, such as succinate dehydrogenase, succinyl Co‐A synthetase leading to excessive generation of succinate.

The lactate levels at baseline were found to be significantly elevated in Group 1 (*p* < 0.02) and Group 2 (*p* < 0.003) as compared to Group 0, indicating the role of lactate in the maintenance of energy. After 6 months, the lactate levels declined in Groups 1 and 2 compared to Group 0 but were not significant. Thus, there was a positive correlation of lactate levels with ASCI, inversely related to recovery. Lactate levels usually rise for a short time during any ischemic injury rising within 5 min after injury and declining by 48 h due to anaerobic glucose degradation. However, in the case of ASCI, as there was a continuous requirement of energy, lactate levels remain elevated, acting as a secondary reservoir of energy. The raised lactate levels were also responsible for muscle paralysis.

The rest of the two metabolites, acetate in Group 1 and valine in Group 2, decreased significantly (*p* < 0.05) from baseline to 6 months.

Thus, to summarize the cited study, the stem cell‐treated group had better neurological recovery outcomes than the fixation alone group, indicating their neuroprotective property. The observations also showed the various injury mechanisms and on‐going inflammatory activity in patients with ASCI. It was concluded that neuroregeneration through the stem cells was responsible for the healing process after 6 months.^39^

Another prospective case‐control study was conducted by Singh et al. with ASCI subjects of age group 18–65 years old, in which 140 subjects were subdivided into three groups. The first group (Group 1) was healthy controls (n = 70); the second group (Group 2) was the stem cell fixation group (n = 35) and the third group (Group 3) was the fixation alone group (n = 35). The treatment in Group 2 included posterior instrumentation with augmentation of autologous bone marrow‐derived mononuclear cells, while Group 3 included treatment with only posterior instrumentation. The urine samples were collected in two phases: Phase 1 included just after the admission (baseline), 6 weeks after surgery, after 3 months and Phase 2 after 6 months follow‐up. The pH of urine samples was maintained at 7.4 with phosphate buffer, and ^1^H NMR spectra were acquired at 800 MHz using one‐dimensional Nuclear Overhauser Enhancement Spectroscopy pulse sequence with pre‐saturation. The serum collection was also performed at the third and sixth months of the follow‐up study. Multivariate group‐wise analyses were used to assess the severity of cases with metabolic alterations. The metabolites which were identified in the baseline (just after the admission) included β‐hydroxybutyrate, alanine, creatine, creatine phosphate, creatinine, glucose, propylene, phenylalanine, urea and glycol whereas, the metabolites which were identified in the 6‐month follow‐up study included creatine, acetate, creatinine, urea and phenylalanine. It was reported that the elevations in the levels of alanine, creatine, creatine phosphate, creatinine, phenylalanine, glucose, urea and propylene glycol were associated with the ASCI pathogenesis. The metabolites alanine, β‐hydroxybutyrate, acetate, choline‐containing compounds, phenylalanine, creatine, creatinine, creatine phosphates, urea and propylene glycol were found to be associated with ASCI recovery. In Groups 2 and 3, neurological scores were significantly improved at the sixth week (*p* = 0.009) and third month (*p* = 0.043) compared to their baseline values, while they were insignificant compared to 6‐month follow‐up values. The neurological recovery was observed only in the stem cell fixation group, which was Group 2. It was concluded from metabolic alterations that neurological recovery could be easily prognosticated for the ASCI. The variable importance in projection (VIP) scores were derived from OPLS‐DA models to validate the significance of the results. The VIP > 2 (propylene glycol, ß‐hydroxybutyrate, alanine); VIP > 4 (creatine phosphate, creatinine); VIP > 5 (glucose); VIP > 6 (urea),and VIP > 7 (phenylalanine), were derived from OPLS‐DA models between healthy controls and ASCI group at baseline. After 6 months of follow‐up, the OPLS‐DA model between healthy controls and ASCI group indicated VIP > 2 for acetate; VIP > 4 for creatine phosphate, creatine; VIP > 6 for urea and VIP > 7 for urea phenylalanine.

The authors concluded from the present study that surgical therapy significantly contributed to the overall ASCI management, whereas stem cell therapy augmented neurological recovery. Also, the severity of damage in ASCI patients was correlated with metabolic alterations in urine.^40^

### 2.2 Amyotrophic lateral sclerosis

Amyotrophic lateral sclerosis (ALS) is a neurodegenerative disease that affects the motor neurons of the human motor system by degenerating cortical and spinal motor neurons. The disease is of two categories, sporadic and familial, with an incidence of 1 in 1,00,000.^41^, ^42^ The pathophysiology is aberrant axonal transport, glutamate excitotoxicity, oxidative stress, mitochondrial dysfunction, apoptosis and microglial activation. The gene which encodes copper‐zinc superoxide dismutase‐1 is also associated with ALS.^43^ The prevalence of ALS in India is reported to be 3–5 per 1,00,000 populations, with an annual incidence of 1.5‐2.7 per 1,00,000 populations. The average age of onset for the Indian population lies between 55 and 65 years, with a male preponderance at a ratio of 1.5:1.^44^, ^45^

Metabolomic analysis of serum samples was performed in 30 ALS patients (25 males and 5 females) and 25 healthy controls using NMR spectroscopy at 400 MHz.^46^, ^47^, ^48^ One‐dimensional ^1^H NMR spectra were acquired with CPMG sequence with water pre‐saturation at 298 K. The typical parameters used were: data points = 32 K; the number of scans = 128; spectral width = 8000 Hz; relaxation delay = 15 s. The T2 filtering with an echo time of 640 µs repeated 420 times was used, resulting in a total duration of effective echo time as 269 ms. A total of 16 metabolites were significantly altered in ALS patients as compared to healthy controls. These metabolites were: branched‐chain amino acids, β‐hydroxybutyrate, lactate, alanine, lysine, acetate, acetone, pyruvate, glutamine, glutamate, citrate, creatine/creatinine, glucose, histidine, tyrosine and formate. Out of these 16 metabolites, only six metabolites were significantly altered as compared to healthy controls. The levels of acetate (*p* < 0.01), β‐hydroxybutyrate (*p* < 0.001), formate (*p* < 0.001) and acetone (*p* < 0.05) were significantly elevated in ALS patients relative to healthy control subjects. On the other hand, serum concentrations of histidine (*p* < 0.001) and glutamine (*p* < 0.02) were significantly reduced in ALS patients compared to healthy control subjects.

Glutamate is a bioenergetic substrate, a non‐essential amino acid that plays a vital role in normal and neoplastic cell differentiation. It actively participates in biosynthetic, metabolic, bioenergetic and oncogenic signalling pathways. It is a powerful neurotransmitter that plays an important role in the formation of synapses and neurons. It causes neurotoxicity in ALS through its excitotoxic mechanisms, which can further lead to cell death.^49^, ^50^, ^51^ In the present study, a significant elevation of glutamate was observed in ALS patients compared to the control group. Excitotoxicity mechanisms were consistent with ALS pathogenesis with significantly elevated glutamate levels. On the contrary, glutamine concentrations were decreased in ALS patients compared to the control group. It was concluded that disturbance during glutamate‐glutamine conversion might lead to excitotoxic effects in astrocytes and postsynaptic neurons.

Another metabolite, histidine, was found to be significantly decreased in ALS patients. Histidine is known as reactive oxygen species (ROS) scavenger due to the presence of imidazole in its structure which depicts an anti‐inflammatory response.^46^ In an experimental study, histidine was reported to have a neuroprotective effect against hypoxic and oxidative injury.^52^ In the ischemic hemisphere, histidine preserved superoxide‐dismutase activity through endogenous scavenging preservation. It has been reported that a lower concentration of histidine was found to be associated with inflammation, energy wasting and oxidative stress.^53^

The levels of formate were also elevated in the ALS patients compared to the healthy controls. Formate is a by‐product of acetate. Normal levels of formate help to maintain cellular function and protect from cell death by inhibiting cytochrome oxidase enzymatic activity and maintaining sufficient levels of adenosine diphosphate (ATP).^54^ Elevated levels of formate lead to metabolic acidosis, disruption of mitochondrial electron transport chain and excessive inhibition of cytochrome oxidase activity. This inhibition of cytochrome oxidase may lead to cell death due to the formation of ROS, leading to the blockade of the electron transport chain. It was documented that the elevated levels of formate in ALS patients indicated that toxic effects might be mediated through oxidative stress.

The levels of ketone bodies (acetone and β‐hydroxybutyrate) were found to be elevated in the ALS patients. The higher energy demand might have been met in the disease state by enhanced levels of such ketone bodies. In different models of Alzheimer's and Parkinson's, β‐hydroxybutyrate has been suggested as neuroprotective.^55^ The levels of antioxidant glutathione have been reported to be reduced due to enhancement of oxidative stress in the ALS patients; in such patient's neuroprotective effect of β‐hydroxybutyrate has been well documented. Further, the acetate levels were elevated in ALS patients. Acetate helps in maintaining adequate amounts of ATP in normal cells.

Based on the metabolic changes, it was inferred that an imbalance in the peripheral metabolic concentration might direct similar changes in the CNS. The excitotoxicity of glutamate leads to ROS formation, which can further lead to an imbalance in the oxidant‐antioxidant ratio, reduction in the histidine concentration, enhancement in the β‐hydroxybutyrate and acetone. ROS can lead to the accumulation of formate, which can further interrupt the mitochondrial electron transport chain. The current study revealed abnormal metabolic alteration patterns which could have the potential to serve as biomarkers for monitoring ALS disease progression.^56^

### 2.3 Tethered cord syndrome

Tethered cord syndrome is a neurological disorder that arises from spinal cord fixation due to limited spinal cord movement.^57^ Continuous stretching and enhanced spinal cord tension in a child potentially lead to several neurological disorders and other symptoms. Different types of tethered cord syndromes which may be acquired or congenital include lipomeningomyelocele (LMMC), diastematomyelia, dermoids, filum terminale and dermal sinus tracts.^58^ The majority of disorders depend upon the progressive involvement of fibrosis into the filum terminale. The spinal cord tip and sacrum (tailbone) provide strand to the filum terminale to its embryonic evolution. During embryonic evolution, the defective termination in the neural tube results in the inflexible structures in children, which leads to spina bifida. Tethered cord syndrome can be treated through early age surgery.^57^, ^58^

The congenital or primary syndrome is associated with spina bifida. Spina bifida is a birth defect due to incomplete closure of the bony vertebral arch or lamina and posterior spinal cord. Acquired or secondary cause includes tumours, surgery or infections, or fibrosis evolvement to the spinal cord. Complications of spinal surgery may also develop tethered cord syndrome.^59^ According to Allagh et al., the overall pooled birth prevalence of tethered cord syndrome, also known as neural tube defects, has been reported in 4.5 per 1000 populations of total births.^60^ Pathophysiological mechanisms include inevitable utilization of oxygen by the spinal cord due to impaired oxidative metabolism. Ischemic effects may disrupt the oxygen supply as well as ionic channel dysfunction. Such impairment may cause a straightening of the spinal cord. During gestation, the spinal cord in the form of neuronal fibres (axons) is constant to the brain and spinal canal (tailbone area). If the posterior spinal cord is shackled and unable to grow with a vertebral column in childhood, the extension of the spinal cord takes place far off than physiological tolerance.^61^

In a study by Sharma et al., cerebrospinal fluid (CSF) samples were analysed for pre‐ and post‐operative levels of metabolites by ^1^H NMR spectroscopy. The study subjects included 16 infants and children suffering from spinal dysraphism and 10 age‐matched children as control. The study subjects were divided into pre‐operative groups, including spinal dysraphism and tethering of the cord and post‐operative including post‐operative spinal dysraphism and untethering groups. Spinal dysraphism group with tethering of the cord, a low‐lying cord or, a second lesion included six patients. Four were with meningomyelocele (MMC), while two were with LMMC. Retethering of the cord group included 10 children who underwent surgery earlier but were found with tethering. In this group, four patients had an MMC with a tethered cord, two patients had a tethered low‐lying fatty filum and four had LMMC with a low‐lying cord. Patients in the post‐operative spinal dysraphism group underwent laminectomy, excision and untethering with or without evidence of excision of a second lesion. In the untethering group, six patients were found to have signs of tethering, among which two patients were with a tethered low‐lying fatty filum and four were with an MMC and a tethered cord. Two months post‐operatively, CSF samples were analysed for metabolic alterations using ^1^H NMR spectroscopy at 400 MHz using a single pulse sequence.^62^, ^63^

The levels of lactate, alanine, acetate, choline and glycerophosphocholine were found to be significantly altered among all the groups. In pre‐operative spinal dysraphism group, the levels of lactate, alanine and acetate were found to be significantly elevated (*p *< 0.001) as compared to the control group. In the post‐operative spinal dysraphism group, levels of only alanine (*p* = 0.04) were significantly decreased as compared to the control group. In pre‐operative tethering group, levels of lactate (*p* = 0.02), alanine (*p* = 0.02), acetate (*p* = 0.003), choline (*p* = 0.003) and glycerophosphocholine (*p* = 0.001) were significantly elevated in comparison to control group. In post‐operative spinal dysraphism, the levels of lactate (*p* < 0.001), alanine (*p* < 0.001), acetate (*p* = 0.001), choline (*p* = 0.03) and glycerophosphocholine (*p* = 0.02) were significantly decreased compared to pre‐operative spinal dysraphism group. In tethering group, the levels of acetate (*p* = 0.01), choline (*p* = 0.02) and glycerophosphocholine (*p* = 0.002) were significantly decreased compared to untethering group. In tethering versus spinal dysraphism group, levels of lactate (*p* = 0.001) and alanine (*p* < 0.001) were significantly elevated in spinal dysraphism.^64^, ^65^, ^66^, ^67^, ^68^, ^69^

Lactate is a by‐product of glycolytic metabolism; levels are generally increased in response to ischemia of the brain. The process of glycolysis plays a vital role in cerebral microcirculation at oxygen‐deprived regions with acute trauma and specific physiological imbalances.^63^ The enhanced level of pyruvate and plasma lactate was also observed in the case of mitochondrial dysfunction, which represents the metabolic disorders with the nutritional deficiency of thiamine.^64^, ^65^ The enhanced level of lactate can also be seen in several central nervous system's disorders like stroke, inflammation, malignancy and seizures.^64^

Acetate plays a significant role in lipogenesis and protein acetylation. Acetate is quantitively generated by pyruvate, the end product of glycolysis and a key node in central carbon metabolism. During nutritional excess, such as hyper‐active glucose metabolism, the phenomenon of acetate formation becomes more pronounced. This formation of pyruvate to acetate might lead to the formation of ROS and pyruvate decarboxylation through keto acid dehydrogenases. The elevation of acetate metabolite enhances the anaerobic respiration or oxidative impairment, which results in spinal cord dysfunction. Tissue anoxia leads to anaerobic metabolism in CSF with the elevation of lactate and acetate.^70^ Alanine is also a biomarker for mitochondrial dysfunction for certain conditions like regression or physiological stress. OXPHOS disease where there is a defect in oxidative phosphorylation may be attributed to an alteration in the incorporation of alanine. The levels of creatine, phosphocreatine and glucose were normal in both pre‐and post‐operative groups. Choline and glycerol phosphorylcholine levels were also elevated in patients before surgery, which was reversed after the surgery. Glycerol phosphorylcholine is impermeable to the cell membrane after its formation, which may lead to elevation levels in the CSF.^68^

### 2.4 Spina bifida

Spina bifida is a birth defect associated with incomplete closure of the bony vertebral arch or lamina and posterior spinal cord. Due to connectivity impairment in the dermal sinus tract and caudal spinal cord, it is interlinked with the tethered cord syndrome. The worldwide occurrence rate is reported to be 1 of 1000 births.^71^ Lipomyelomeninocele is another fatty anomaly in which the spinal canal expresses lipoma underneath the spinal cord linings. The clinical subtypes are myelomeningocele which is lumbosacral spinal tube failure at the embryonic stage. The genetic cause of myelomeningocele was estimated to be about 60–70%. Non‐genetic risk factors include anticonvulsant therapy, folate intake reduction, obesity and diabetes mellitus. It can affect the lifestyle of all age groups of individuals, which may challenge the quality of life of society as well as families.^72^ In India, the prevalence of spina bifida has been reported to be 0.19% of births, approximately ranging from 0.14 to 0.27%.^73^

In a study by Pal et al., the metabolomic profiling of CSF was performed in patients suffering from spina bifida by NMR spectrometry at 400.13 MHz using a single pulse sequence. All 16 patients (including infants and children) were included, in which six patients were cases of MMC, and 10 were in spina bifida with tethering of cord. Age‐matched 10 children were categorised into a control group. There was a significant elevation of metabolites in MMC group as compared to control group including acetate (*p *< 0.001), lactate (*p* < 0.001), choline (*p* = 0.002), glycerol phosphorylcholine (*p* < 0.002), alanine (*p* < 0.001); whereas significantly elevated values of metabolites such as acetate (*p* < 0.003), lactate (*p* < 0.022), choline (*p* < 0.033), glycerol phosphorylcholine (*p* < 0.003) were observed in tethering group relative to control group. The glucose and creatinine levels remained unaltered in both MMC and tethering groups compared to the control group.^74^ It was concluded from the study that metabolic aberrations were associated with the pathogenesis of spina bifida. The specific biomarkers proposed included creatine in neuronal damage, alanine and phenylalanine in ischemia, lactate and acetate in anaerobic metabolism and choline in cell membrane dysfunction.^71^, ^75^, ^76^, ^77^, ^78^, ^79^

### 2.5 Stroke

Stroke is the most devastating neurological condition standing in fifth place for mortality after heart, cancer, chronic lower respiratory disease and unintended injuries or accidents.^80^ The prevalence of stroke in India varies from 44.29 to 559/1,00,000 people. Its incidence ranges from 105 to 152 per 1,00,000 persons per year, as estimated for two decades.^81^ The complex array of ischemic‐reperfusion injury mainly involves adenosine triphosphate depletion, lactic acidosis, ion homeostasis disruption, including calcium and sodium overload, enzyme induction predominantly proteases, phospholipases and endonucleases, oedema, mitochondrial dysfunction, glutamate excitotoxicity, oxidative stress, cortical spreading depression, inflammation, leading to apoptotic and necrotic cell death.^82^, ^83^ Currently available limited unsatisfactory therapeutic options for acute stroke, include intravenous recombinant tissue plasminogen activator, with a narrow therapeutic time window of only 4.5 h along with a risk of haemorrhagic transformation and intra‐arterial devices initiation within 6 h after stroke onset.^84^, ^85^ Particularly, the primary goal of post‐stroke treatment is the protection of the penumbral region from tissue injury. Balancing of altered brain metabolites is the scope for the development of novel treatment strategies for stroke.^86^

Studies published by Chauhan et al., Gupta et al. (2018), and Gupta et al.(2020) had evaluated the effect of neuroprotective agents on neurometabolic alterations in the middle cerebral artery occlusion (MCAo) model of stroke in rats by ^1^H NMR technique. In all the above‐mentioned studies, rat brain cortex tissues were processed by the perchloric acid extraction method. The spectra were acquired using 700 MHz. NMR spectrometer and single 60̊ pulse (Ernst angle) sequence with water suppression. Principal component analysis (PCA) and partial least squares discriminant analysis (PLS‐DA) was performed using Unscrambler 10.2 (Camo). The variables were mean‐cantered and unit variance scaled, followed by PLS‐DA using seven components. Similarities and differences between groups were identified by component plots analysis. Random permutation of the response variable and comparison of the goodness of fit (R2Y and Q2) from PLS‐DA analysis were used for validating statistical models.^87^, ^88^, ^89^

As reported by Chauhan et al., the neuroprotective effect of rapamycin was found to attenuate metabolic perturbations in the MCAo model of transient focal cerebral ischemia in rats. In this study, 44 neurometabolites were assigned and out of these nine metabolites were found to be significantly altered in vehicle‐treated MCAo control group (*p* < 0.05) in comparison to vehicle‐treated sham group estimated 24 h post‐MCAo. Out of these nine neurometabolites, the levels of lactate were found to be elevated, while levels of choline‐containing compounds, taurine, glutamate/glutamine, creatine/phosphocreatine (Cr/PCr), GABA, myo‐inositol, glycerophosphocholine and NAA were decreased in vehicle‐treated MCAo control group in comparison to vehicle‐treated sham group. Rapamycin‐treated rats showed significant neuroprotective effect with evidence of a reduction in metabolic alterations attributed to attenuation of glutamate‐induced neurotoxicity, calcium homeostasis and cell membrane metabolism.^87^

In another study by Gupta et al., the neuroprotective effect of lercanidipine in MCAo‐induced cerebral ischemic‐reperfusion injury was studied by metabolomics approach. Rat brain cortex tissue collected 24 h post‐MCAo from sham, MCAo control and lercanidipine‐treated rats were subjected to perchloric extraction. These tissue extracts were analysed by ^1^H NMR‐based metabolomics. The data revealed significant alterations in 23 metabolites after cerebral ischemic‐reperfusion injury. The treatment with lercanidipine significantly attenuated the five neurometabolites (*p* < 0.05) levels, including tyrosine, alanine, valine, acetate and lactate, which were elevated in the MCAo control group. On the other hand, the remaining 10 neurometabolites, adenosine‐diphosphate, taurine, myoinositol, NAA, glutamate, glutamine, aspartate, choline, Cr/ Cr/PCr and glycerophosphocholine were elevated in the lercanidipine‐treated group (*p* < 0.05) as compared to the MCAo control group. The rest of the eight neurometabolites, including histidine, formate, tryptophan, phenylalanine, glycine, γ‐aminobutyric acid, leucine and isoleucine, remained unaltered in the lercanidipine‐treated rats as compared to the MCAo control group. It was concluded that the neuroprotective effect of lercanidipine might be mediated through attenuation of oxidative stress, bioenergetics, amino acid metabolism, nucleotide metabolism, mitochondrial dysfunctions and inflammation.^88^

Similarly, in continuation with a series of experiments by Gupta et al., citalopram, an antidepressant which is a selective serotonin reuptake inhibitor was found to be neuroprotective in the MCAo model of transient cerebral ischemia in rats.^89^ Metabolomics analysis was performed using ^1^H NMR spectroscopy at 700 MHz in rat brain cortex tissue samples. The citalopram‐treated group (administered at two points: 1 h prior to MCAo and 0.5 h post‐reperfusion) depicted alteration in 17 metabolites as compared to vehicle‐treated MCAo control group. The treatment with citalopram significantly elevated the levels of NAA (*p* < 0.0043), Cr/PCr (*p* < 0.0087), choline (*p* < 0.0411), glycine (*p* < 0.0260), myo‐inositol (*p* < 0.0260), aspartate (*p* < 0.0087), glutamate (*p* < 0.0087), glutamine (*p *< 0.0152), taurine (*p* < 0.0152) and adenosine diphosphate (*p* < 0.0087). While treatment with citalopram significantly decreased the levels of leucine (*p* < 0.0260), valine (*p *< 0.0411), alanine (*p* < 0.0260), lactate (*p* < 0.0260), acetate (*p* < 0.0303), tyrosine (*p* < 0.0022) and phenylalanine (*p* < 0.0260) levels as compared to MCAo control group. It was inferred from the study that citalopram neuroprotective effect was mediated through amelioration of oxidative stress, inflammation and metabolic alterations.^89^

### 2.6 Parkinson's disease

Parkinson's disease (PD) is a progressive neurological disorder involving the nigrostriatal dopaminergic pathway. It is characterised by dopamine depletion in the striatum. The disease is associated with a broader range of symptoms with unknown aetiology and insufficient therapeutic options.^90^ The prevalence of PD in India was estimated to be 10% of the global burden with a projection of 5.8 lakhs cases, while crude prevalence rates were between 6 and 53 of 1,00,000 in 2016.  The prevalence rates were age related, with an incidence of 247 per 1,00,000 in people above 60 years and approximately 1% in those above 65 years.^91^

In a study by Kumari et al., metabolic profiling of saliva from PD patients (n = 76) and healthy volunteers (n = 37), 40 metabolites were identified using ^1^H NMR spectroscopy at 700 MHz. The OPLS‐DA revealed a significant separation between salivary samples of PD and HC groups, with predictive capabilities as R2X = 0.10; R2Y = 0.56, Q2 = 0.42. Significantly elevated levels of metabolites: trimethylamine *N*‐oxide (*p* < 0.016), GABA (*p* < 0.019), *N*‐acetyl glutamate (*p* < 0.006), acetoin (*p* < 0.001), phenylalanine (*p* < 0.005), tyrosine (*p* < 0.016), histidine (*p* < 0.003), glycine (*p* < 0.023), fucose (*p* < 0.019), propionate (*p* < 0.001), acetoacetate (*p* < 0.047), taurine (*p* < 0.061), valine (*p* < 0.010), isoleucine (*p* < 0.006), alanine (*p* < 0.019) and acetate (*p* < 0.027) that were observed in PD patients as compared to healthy volunteers. It was concluded from this study that metabolic alterations in energy, amino acid, ketone bodies and gut microflora pathways were found to be involved in the PD pathogenesis. Saliva may be a better diagnostic medium as is physiologically equivalent to serum in the discovery of biomarkers for neurological disorders.^92^

In another study published by Kumari et al., metabolic profiling of urine samples for idiopathic PD (IPD; n = 100) patients and healthy controls (HC; n = 50) were performed at 700 MHz. The levels of six metabolites, including ornithine (*p* < 0.00001), phenylalanine (*p* < 0.0010), tyrosine (*p* < 0.0065), isoleucine (*p* < 0.0022), β‐hydroxybutyrate (*p* < 0.0047) and succinate (*p* < 0.0075) were found to be elevated in IPD patients in comparison to HC group. The receiver operating characteristics curve analysis showed an area under the curve greater than 0.60 for these metabolites. A positive correlation was found between succinate concentration and unified Parkinson's disease rating scale scores.^93^

In another study by Nagesh et al., ^1^H NMR spectroscopy at 800 MHz was used to study the metabolic alterations in serum of 22 healthy controls, 17 patients suffering from PD, along with seven patients associated with progressive supranuclear palsy (PSP) and six patients with multiple system atrophy (MSA). The metabolites that were elevated in PD, PSP and MSA as compared to control groups were: glutamate and glucose in PD (*p* < 0.001), PSP and MSA (*p* < 0.05); glutamine, alanine, valine, isoleucine, histidine, lactate, acetone and formate in PD, PSP and MSA (*p* < 0.001); citrate and threonine in PD, PSP and MSA (*p* < 0.05). It was inferred that branched‐chain amino acids mainly contributed to the pathogenesis of PD mediated through increased neuronal excitability.^94^

Paraquat (PQ), an environmental toxin widely implicated as an herbicide, has been associated with PD pathogenesis. Metabolic alterations in PQ‐induced PD model in *Drosophila melanogaster* flies and control flies were investigated by GC‐MS technique using Trace GC ultra (Thermo Scientific, FL, USA) coupled with TSQ Quantum XLS mass spectrometer (Thermo Scientific). The flies were exposed by feeding them in the diet with PQ at three different concentrations as 5, 10 and 20 mM for 12 and 24 h. PCA identified the systematic variations and general clustering behaviour among all the groups. Differential metabolites were identified for the separation between groups using PLS‐DA. Further, VIP scores were derived, representing the relative contribution of individual metabolites to the variance among the control and PQ‐exposed groups. The GC‐MS metabolomics revealed that alterations in 24 metabolites indicated PQ‐induced oxidative stress, behavioural alterations associated with movement disorders and dopaminergic neurodegeneration. The levels of several metabolites significantly altered in brain samples of PQ exposed as compared to the control *D. melanogaster* group: VIP > 2 was seen for cis‐9‐hexadecenoic acid, myoinositol phosphate, phosphoric acid, glycerol, 9, 12‐octadecadienoic acid, glycine, hexadecanoic acid, oleic acid, leucine, uric acid, ethanolamine, alanine, 1‐monostearate, valine, 1‐monopalmitin, lactic acid, isoleucine, glucose, inositol and eicosanoic acid; VIP > 3 was observed for galactose, trehalose and VIP > 4 was obtained for GABA, proline. The changes in metabolites levels were expressed in fold change in brain samples of the PD model compared to the control group. The levels of GABA, proline, galactose, trehalose, leucine, uric acid, valine, isoleucine and glucose, were decreased. In contrast, increased levels of cis‐9‐hexadecenoic acid, myoinositol phosphate, phosphoric acid, glycerol, 9, 12‐octadecadienoic acid, glycine, hexadecanoic acid, oleic acid, ethanolamine, alanine, 1‐monostearate, 1‐monopalmitin and lactic acid were observed in the PD model compared to the control group.^95^

In another PD model of rotenone (ROT) induced neurodegeneration in *D. melanogaster*, the anti‐Parkinsonian activity of (4‐(5‐phenyl‐3‐3‐3‐(4‐ trifluoromethyl‐phenyl)‐ureido‐propyl‐pyrazole‐1‐yl)‐benzenesulfonamide) (PTUPB), a dual inhibitor of soluble epoxide hydrolase and cyclo‐oxygenase‐2 was investigated. The study was designed with two objectives: the first one was to estimate the bioavailability of PTUPB in the fly heads and another one was neuroprotective effects of PTUPB in ROT‐induced dopaminergic neurodegeneration in *D. melanogaster*. For the purpose, two separate LC‐MS/MS methods were developed that included: Method‐I (PTUPB estimation) to determine the PTUPB bioavailability in fly heads and Method‐II (Neuroprotection study) including estimation of levels of dopamine and its metabolite 3,4 dihydroxy phenylacetic acids (DOPAC) upon administration of PTUPB at 100 and 250 µm doses. For Method I, 50 flies were investigated for bioavailability of PTUPB in fly heads upon administration of 100 and 250 µm doses orally. The fly heads were isolated at three different time points at 0.02th, 4th and 7th days after PTUPB administration. In Method‐II, 50 flies were subdivided into four groups: Group 1, administered vehicle as dimethyl sulphoxide (0.25% v/v); Group 2, control, administered sucrose solution (7% v/v); Groups 3 and 4 were treated with PTUPB at a dose of 100 and 250 µm, respectively. Either vehicle or PTUPB treatments were initiated 4 days prior to ROT (500 µm) treatment. Except for Group I, all the groups received ROT on the fifth day of sucrose or PTUPB treatment. The heads were isolated on the 12th day for the estimation of dopamine and DOPAC. The ultraforce LC system coupled with tandem quadrupole mass spectrometer (Shimadzu 8030, Tokyo, Japan), equipped with an interface of electrospray ionization, SIL20AC autosampler, SPD‐M20 PDA detector, CTO‐20AC column oven, LC‐20AD pump and CBM‐20 Elite controller, was used for method validation and estimations. The chromatographic separation method was validated on Jones (50 × 4.6 mm; 3 µ) column at an ambient temperature using isocratic elution solutions including formic acid (0.1% v/v) and acetonitrile (20:80 v/v) at a flow rate of 0.4 mL/minfor Method‐I and acetic acid (0.1% v/v) and methanol at a flow rate of 0.5 mL/min for Method‐II. For Method‐I, celecoxib was used as internal standard 1 along with main working parameters as heat block temperature of 350℃ and desolvation line temperature of 250 ℃; capillary voltage of 1.3 kV; ultrapure nitrogen gas was used as nebulizing gas at a rate of 3 L/min and drying gas at a rate of 15 L/min. Collision‐induced dissociation experiments were performed using ultrapure argon gas at 230 kPa. For monitoring daughter ions, the mass spectrometric acquisitions of analyte signals were performed in positive and negative electron spray ionization using Multiple reaction monitoring modes. For Method II, l‐phenylalanine was used as IS‐2 with all the working parameters as Method‐I. In the present study for Method I, dose and time‐dependent (0.02 to 7 days treatment) increase in PTUPB concentration were observed in the range from 4.99 to 17.64 ng/fly and 6.65 to 19.64 ng/fly at the doses 100 and 250 µm, respectively. In Method‐II, the dopamine and DOPAC levels were significantly decreased in control (Group 2) as 0.15 ± 0.012 ng/mg protein and 64.00 ± 2.75 ng/mg protein as compared to normal group (Group 1) as 1.36 ± 0.043 ng/mg protein and 96.51 ± 1.35 ng/mg protein, respectively, depicting neurodegenerative effects. Dose‐dependent neuroprotective effect of PTUPB was observed against ROT‐induced PD model (Groups 3 and 4 vs. Group 2) as indicated by the significantly elevated levels of dopamine and its metabolite DOPAC.^96^

## 3 GLIOBLASTOMA

The most frequent and aggressive primary malignant brain tumour in the adult population is Glioblastoma multiforme (GBM; WHO grade IV astrocytoma). The standard treatment is surgery followed by radiotherapy and adjuvant chemotherapy with a median overall survival between 10 and 20 months.^97^ The incidence of GBM tumours in India is 1 to 4 per 1,00,000 evident usually in the fifth and sixth decades of life.^98^

In a study by Jothi et al., metabolic alterations in low‐grade (LG) and high‐grade (HG) glioblastomas were investigated by ^1^H NMR spectroscopy at 500 MHz. These glial tumour samples were categorised as pilocytic astrocytoma as Grade I (n = 3), diffuse astrocytoma as Grade II (n = 3), anaplastic astrocytoma as Grade III (n = 3) and Glioblastoma Multiforme as Grade IV (n = 4). For the control group, the age‐matched non‐malignant histologically confirmed patients were considered (n = 3). Discrimination of metabolites was performed using the two‐class model (LG and HG) by PLS‐DA and OPLS‐DA. The two‐class model robustness was checked using a permutation test (1000 cycles) at *p* < 0.004 and cross‐validation included R2 (cum) = 0.945, Q2 (cum) = 0.873. Out of 35 metabolites, the top 15 metabolites differentiating the metabolomics of LG and HG were considered and ranked as per their VIP scores. These metabolites included taurine (*p* < 0.0001), creatine (*p* < 0.0001), lactate (*p* < 0.0001), glutamine (*p* < 0.0001), GABA (*p* < 0.0003), glutamate (*p* < 0.0004), phenylalanine (*p* < 0.0005), glycerol phosphocholine (*p* < 0.0016), valine (*p* < 0.0011), l‐myoinositol (*p* < 0.0018), isoleucine (*p* < 0.0196), choline (*p* < 0.0396), glutathione (*p* < 0.0155), tyrosine (*p* < 0.0120) and leucine (*p* < 0.0293). The VIP score >2 included glycerol phosphocholine, valine, l‐myoinositol, isoleucine, choline, glutathione, tyrosine and leucine; VIP score > 4 included creatine, lactate, glutamine, GABA, glutamate, phenylalanine and VIP score > 5 included taurine. It was concluded that with specific grades of gliomas the pattern of metabolic alterations was reasonably consistent which would help in optimizing the treatment strategies.^99^

### 3.1 Neurological disorders with inborn errors of metabolism

Inborn errors of metabolism (IEM) comprise a group of over 500 heterogeneous disorders resulting from a defect either in the breakdown or storage or enzymes involved in carbohydrates, fatty acids and protein intermediate metabolic pathways.^100^ IEM is broadly classified into amino acid disorders (homocystinuria and homocystinuria), organic acid disorders (propionic aciduria, methylmalonic aciduria, isovaleric acidemia, biotinidase deficiency), fatty‐acid disorders (short‐ or medium‐chain acyl‐coenzyme A dehydrogenase deficiency), lysosomal storage disorders (sphingolipidoses such as Fabry, Farber, Gaucher and Niemann–Pick diseases, mucolipidosis, oligosaccharides), carbohydrate metabolism disorders (galactosemia, Pompe's disease), urea cycle disorders (citrullinemia, arginine mix), mitochondrial disorders (Leigh syndrome) and peroxisomal disorders (Zellweger syndrome, Refsum syndrome). Further, miscellaneous IEM includes purine and pyrimidine disorders, mental disorders, porphyria, haematological disorders, lipid disorders and myelin metabolism disorders.^101^ These malformations are clinically concerning. These may delay attaining developmental childhood milestones, lead to acute decompensation in the neonatal period and non‐specific manifestations such as severe low blood sugar, acute metabolic crisis, metabolic acidosis, epilepsy and high blood ammonia.^102^

The worldwide prevalence of IEM is reported as 50.9 per 1,00,000 live births and a minimum of 23,529 deaths per year. In India, the prevalence of IEM has been reported to be 1 in 2497 newborns.^103^ Annually, approximately out of 24 million newborns, 3,40,000 are born with glucose‐6‐phosphate dehydrogenase deficiency, 7,80,000 have congenital malformations, 21,000 have Down syndrome, 20,800 have metabolic disorders, 9000 have thalassemia, 10,400 have congenital hypothyroidism and 5200 have sickle cell anaemia.^103^, ^104^ In the present review, only IEM disorders showing clinical manifestations associated with neurological disorders, such as Maple syrup urine disorder (MSUD) with metabolic encephalopathy and citrullinemia with generalised tonic‐clonic seizures and Glutaric acidemia type I with brain atrophy and macrocephaly, have been incorporated.

MSUD is a rare autosomal recessive inherited inborn error caused due to deficiency or decreased activity of branched‐chain alpha‐ketoacid dehydrogenase complex.^105^ As a result, there is a decreased catabolism of branched‐chain amino acids, particularly, leucine, valine and isoleucine. It leads to their accumulation in the blood, which commonly progresses to metabolic encephalopathy if they remain undiagnosed for a longer period.^106^ In a case report by Patil et al., a 17‐day‐old boy was the firstborn of non‐consanguineous parents. He was presented to the outpatient department with a history of fever, poor feed intake and lethargy for 2 days. The boy was born to 26‐year‐old primigravida after conceiving intrauterine insemination. Initially, serial ultrasonography was normal during pregnancy, but oligohydramnios was diagnosed at 36 weeks of gestation with no other associated anomaly. Metabolomic profiling was performed using dried blood spots by tandem MS, and urine by GC‐MS expressed as multiples of median (MoM) values. Tandem MS revealed elevated branched‐chain amino acids: leucine/isoleucine/hydroxyproline as 3930 µmol/L (MoM value 30.94) and valine as 585 µmol/L (MoM value 5.13). On the other hand, GC‐MS revealed increased urinary excretion of 2‐hydroxy isovaleric acid as 2368.53 µmol/L (MoM value 3.11), alloisoleucine‐2 as 670.02 µmol/L (MoM value 2.31), isoleucine‐2 as 665.93 µmol/L (MoM value 2.6), leucine‐2 as 3698.97 µmol/L (MoM value 2.96) and valine‐2 as 1976 µmol/L (MoM value 2.41). The authors concluded that neonatal screening helps in the early diagnosis of IEM, such as MSUD, imparting improved outcomes with therapeutic interventions at the appropriate times. Through new‐born screening with tandem MS using dried blood spots has now been a globally recognised tool, but yet is not mandatory in India. As a consequence, the late presentation of the patient in this case report resulted in the development of severe encephalopathy, which might have been avoided and would have reduced the chances of neurodevelopmental sequelae.^107^

Citrullinemia is a disorder of the urea cycle metabolic pathway. There are two types of citrullinemia: type I or neonatal form and type II or adult form. In type I, there is a deficiency of enzyme argininosuccinate synthetase which catalyses the formation of argininosuccinate from citrulline and aspartate. Deficiency of argininosuccinate synthetase leads to hyper‐ammonemia and early death in type I patients, if left untreated. In type II citrullinemia, there is a deficiency of citrin, a mitochondrial aspartate‐glutamate carrier protein.^108^ In a case report published by Lodh et al., there was a development of citrullinemia (type I), glycaemia with hyper‐ammonemia and generalised tonic‐clonic seizures in a 4‐day‐old baby born by caesarean to second degree consanguineous parents at 36th week. The patient's blood was investigated for metabolic alterations by tandem MS. The levels of citrulline 406 µmol/L (normal levels < 70 µmol/L) and glycine 855 µmol/L (normal levels < 505 µmol/L) were found to be elevated, while levels of arginine were low 7.76 umol/L (normal levels < 132 umol/L). The levels of tyrosine, methionine, ornithine, glucose‐6‐phosphate dehydrogenase, thyroid‐stimulating hormones, biotinidase, galactose, 17‐α‐hydroxyprogesterone and immunoreactive trypsinogen were found to be normal. In the present case report, detailed analyses were not performed due to financial constraints. The authors concluded that early diagnosis with biomarkers could help prevent further complications like cerebral palsy and improve long‐term prognosis by early treatment.^109^

GA1 is rare organic aciduria caused by an inherited deficiency of glutaryl‐CoA dehydrogenase involved in the catabolism of l‐tryptophan, l‐lysine and l‐hydroxylysine. This defect leads to elevated glutaric acid, 3‐hydroxyglutaric acid, glutaconic acid and glutaryl carnitine (C5DC). Untreated patients develop neurological symptoms such as brain atrophy and macrocephaly (at birth or later during infancy). Other symptoms include dystonia, and encephalopathic crisis secondary to striatal injury precipitated by immunizations, infectious diseases or surgery.^110^ Organic acids can be detected by GC‐MS and acylcarnitine by tandem MS.^111^

In a pilot clinical study conducted by Babu et al., a total of 17,100 blood samples were screened from June 2012 to June 2014, consisting of paediatric patients and healthy newborns from all over the country. The subjects were divided into two groups: Group 1 (low risk, n = 8731) comprised of healthy new‐born babies up to 7 days after birth, and Group 2 (high risk, n = 8369) consisted of paediatric patients between 0 and 14 years of age associated with abnormalities, such as macrocephaly, metabolic acidosis, delayed motor skills, dystonia and seizures and if any presence of risk factors such as consanguineous marriage and deceased sibling. The samples were screened for their specificity, sensitivity and usefulness of the tandem MS, particularly, Abbott (AB) Sciex 3200, for GA1 by dried blood spot method. Blood samples were spotted on an S903 Whatman filter paper by heel puncture procedure. Analysis of the acylcarnitine and amino acid profiles of the blood spots were performed after derivatization into butylated esters. The samples were separated by running them into the liquid column and then quantified based upon their charge/mass ratio by MS/MS. In this study, screening by tandem MS revealed an incidence of GA1 as 14 in 17,100 sample subjects. The analysis included first‐tier tests with elevated levels of primary analyte C5DC (>0.15 µM/L) and C5DC/C16 ratio (>0.12). The second‐tier test had the presence of organic acids, mainly 3‐hydroxyglutaric acid and glutaconic acid in urine by GC–MS/MS. Out of these, 14 GA1 presumptive positives, four were healthy new‐born babies between 2 and 7 days of age from Group 1 (low risk), while the remaining 10 were from Group 2 (high risk). These 14 GA1 presumptive positive cases were followed‐up, and the samples were analysed for the presence of organic acids in the urine samples by GC–MS/MS. During the follow‐up study, it was found that four patients died before confirmation of GA1, while other four patients were initiated with the treatment. The other five GA1 patients were initiated with the treatment, but without confirmatory testing. Moreover, 100 cases were found to be falsely positive in which analytes were found to be elevated but without any clinical symptoms of GA1. There was one case that remained undetected and the remaining patient's specimens were found to be normal. A significant correlation between age at the time of diagnosis and outcome (*r* = 0.332) was reported. Tandem MS was found to be more than 93.33% sensitive (confidence interval, CI 67.98–98.89) and more than 99.42% specific (CI 99.29‐99.53) with a positive and negative predictive value as 12.28% (CI 6.88–19.75) and 99.99% (CI 99.97‐100), respectively.^112^

Similarly, two case reports for GA1 were published by Pustin et al. In the first case‐report, a one‐and‐a‐half‐year‐old male child, born of a consanguineous marriage, was presented with macrocephaly, hypertelorism, thin sparse hypopigmented hair, wide nasal root and gross developmental delay with a history of recurrent seizure episodes. In the second case report, a 3‐month‐old male child, also born of consanguineous marriage, was presented with macrocephaly, anterior fontanelle bulges and motor delay. Urine analysis in both cases was performed by tandem MS which reported marked renal excretion of glutaric acid suggestive of GA1.^113^

## 4 SUMMARY

Metabolomics is a rapidly booming field that can impact our understanding of molecular mechanisms of neurological diseases. The above‐studies focus on the broad perspective of metabolomics in neurological disorders in India. However, heterogeneity can be seen between the studies, like samples used in the study (CSF, blood urine), protocols for each biofluid and metabolomics data analysis. Nevertheless, metabolomics provides a deeper understanding of disorders and their biochemical pathways in ASCI, amyotrophic lateral sclerosis, tethered cord syndrome, spina bifida, stroke, PD, glioblastoma and neurological disorders with inborn errors of metabolism. In addition, the diagnostic and prognostic biomarkers will further help provide valuable insight into the treatment, response of the therapy and related variations in the mechanism of the disease. Metabolomics has the potential to accredit the plot to the previous biochemical interference. Hence, this could also be a new opportunity to evolve the anticipating biomarkers that can lead to earlier interventions. It may become an indispensable tool in drug discovery and development as biochemistry is fundamental in providing insights into the drugs’ activity, efficacy and safety profile about neurological disorders.

## 5 FUTURE PERSPECTIVES

Metabolomics of CNS disorders would help in mapping out numerous unexplored perturbed neurotransmitters and signalling pathways. This would make it possible to understand better the intricate mechanistic pathways related to neurological disorders, identify prognostic, surrogate and diagnostic markers and predict metabolic response to therapeutic interventions before or during treatment known as pharmacometabolomics.^114^ Targeted and untargeted metabolomic biomarkers for drug‐induced neurotoxicity would help in predicting early assessment of adverse effects associated with the use of drugs before the occurrence of irreversible toxicity.^115^ Identification and monitoring of treatment at an optimal level based on diagnostic tests are known as theranostics. Neurotheranostics will be used as personalised medicines for neurological disorders, thereby, improving diagnostic and therapeutic clinical outcomes.^116^ Further, computational modelling incorporating multiple data sets is another upcoming yet challenging approach for exploring mechanistic pathways of neurological disorders. The present review highlights important studies conducted in India utilizing the metabolomics approach. It is expected that metabolomics approaches, such as pharmacometabolomics, nanotheranostics, toxic metabolomics will flourish and optimise treatment approaches for neurological disorders in India.

## CONFLICT OF INTEREST

The authors declare no conflict of interest.

## Data Availability

Data sharing does not apply to this article as no new data were created or analyzed in this study.
